# Laboratory platform for monitoring SARS-CoV-2 based on surveillance of influenza and other respiratory viruses in Peru

**DOI:** 10.17843/rpmesp.2022.391.8380

**Published:** 2022-03-31

**Authors:** Rosa Palacios-Salvatierra, Maribel Huaringa-Núñez, Priscila Lope-Pari, Johanna Balbuena-Torres, Nancy Rojas-Serrano

**Affiliations:** 1 Laboratorio de Referencia Nacional de Virus Respiratorios, Centro Nacional de Salud Pública, Instituto Nacional de Salud, Lima, Peru. Laboratorio de Referencia Nacional de Virus Respiratorios Centro Nacional de Salud Pública Instituto Nacional de Salud Lima Peru

**Keywords:** Public Health Surveillance, Health Surveillance, Epidemiologic Surveillance Services, Epidemiological Monitoring, Influenza A virus, Influenza B virus, Molecular Diagnostic Techniques, COVID-19 Testing, Public Health Laboratory Services, National Health Systems

## Abstract

In Peru, the COVID-19 pandemic demonstrated the usefulness of having a structured laboratory surveillance system that has been operational for 22 years, based on influenza surveillance; initially in the form of sentinel units, and later strengthened and innovated, with its own resources and with external support, to provide quality information. Biotechnological advances have been implemented for diagnostic confirmation and the capacity of the national laboratory network has been expanded, maintaining efficiency, considering the diverse and complex realities of each region, and overcoming difficulties regarding communication and articulation between institutions. It is necessary to consolidate this system, with collaborative and coordinated work between its components, boosting its effectiveness and timeliness and promoting genomic surveillance of new viruses and variants, as is currently the case with SARS-CoV-2.

## INTRODUCTION

The rapid spread of the coronavirus that causes severe acute respiratory syndrome (SARS-CoV-2), its high transmissibility, morbidity, mortality, and socioeconomic impact worldwide ^1^, prompted the implementation of constant surveillance systems with molecular testing to confirm suspected cases.

In Peru, at the beginning of the pandemic, this challenge was met by the National Referral Laboratory for Respiratory Viruses (LRNVR) of the National Public Health Center (CNSP) of the National Institute of Health (INS), at the Biomedicine center in Chorrillos, Lima. The LRNVR is a World Health Organization (WHO) collaborating center for laboratory epidemiological surveillance of influenza and other respiratory viruses (ORV) that had initiated the technology transfer (TT) of methods to selected regional referral laboratories (RRL) of the National Laboratory Network (RNL).

**Figure 1:**
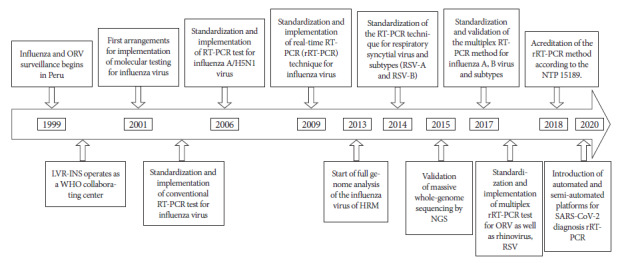
Timeline of the implementation of molecular tests for respiratory virus surveillance in Peru, 1999-2021.

This article compiles information on the implementation of this laboratory surveillance during the last 22 years, with emphasis on the development of molecular tests, reviewing primary and secondary sources such as reports, publications, among others, establishing a sequence of the processes over time (Figure 1).

The aim of this study was to demonstrate that, in the face of the COVID-19 pandemic, Peru had a laboratory surveillance system for detecting new respiratory viruses, new strains and lineages, with molecular testing and genomic sequencing, that is carried out at the INS and in some RRLs, by trained specialists. These capabilities were developed the during influenza virus surveillance and have served as a basis for the challenges of the current pandemic virus.

## BACKGROUND

Laboratory-based respiratory virus surveillance in Peru was established to detect, identify and characterize the etiology, activity and circulation of influenza virus and ORV during cases of severe acute respiratory infections (SARI) and influenza-like illness/flu syndrome (ILI/FS) with the participation of sentinel centers at the national level. Priority was given to the differential diagnosis of influenza virus with ORV, initially adenovirus, parainfluenza 1, 2 and 3, respiratory syncytial virus (RSV), and later rhinovirus and human metapneumovirus. In addition to timely notification, it contributed to developing the influenza vaccine, by detecting the emergence of variants or other emerging pathogens with epidemic or pandemic potential, and technically monitoring the RRLs involved in this surveillance.

The LRNVR has participated in influenza and ORV surveillance since 1999 ^2^ as a WHO collaborating center, sending information through the FluNet computer system to the Center for Communicable Disease Control (CDC) in Atlanta, United States of America (USA), and sending typified positive samples and selected viral isolates for antigenic characterization and molecular genetic study ^3^. Thus, it was possible to confirm the viral etiology of the acute febrile respiratory syndrome and the entry of new influenza strains into Peru (Table 1), at a time when the surveillance systems of developing countries did not have sufficient capacity to do so ^4^.

**Table 1:** Results of laboratory surveillance of influenza and other respiratory viruses in Peru (2000-2020).

Year	Number of samples	Positive %	Influenza A: Types and subtypes	Influenza B: lineages	ORV
2000 ^2^	600	67.6% (406) by IFA and isolation	A(H3N2) 28.3%, similar to: A/H3N2/Panama/2007/99	Flu B: 29,5% B/Beijing Yamanashi/166/98 (early 2000’s), later B/Sichuan/379/99	RSV: 2.6%, parainfluenza: 5.2%, Adenovirus: 2.0%
2001 ^2^ ^,^ ^5^ ^,^ ^6^	1103	85.67% (944) by IFA	A(H3N2) 55 - 60%, similar to: A/H3N2/Panama/2007/99	Flu B: 22% 16.50%: B/Beijing Yamanashi/ 166/98	RSV 2.6-3%, parainfluenza 8.07-11%, adenovirus 3 - 4%
2002 ^2^ ^,^ ^5^ ^,^ ^6^	1327	74.98% (995) by IFA	Influenza A (H3N2/2007/99) 13.94%	Influenza B/ Sichuan/379/99 10.12%	Parainfluenza: 21.55%, RSV: 13.33%, adenovirus: 12%
2004 ^4^	2375	60.8% (1444)	Influenza A 31.65% (457)	Influenza B: 10% (144)	Adenovirus: 18% (261) parainfluenza 10.2% (147) RSV: 30.12% (435)
2005 ^4^	87 (January 2-15)	56%	Influenza A/H1 38% Influenza /H3 32%	Influenza B 2.9% Influenza B: 1.9%	Adenovirus: 1^st^ week: 35%, 2^nd^ week: 3.77% RSV: 1^st^ week: 5.8%, 2^nd^ week: 1.9%
2006-2008 ^11^ ^,^ ^26^	6835	January - February 2006 Surveillance: 37.23% of positives by IFA	Influenza A 25% For 2006: similar to A/H1N1/Solomon Island/03/06 and similar to A/H1N1/New Caledonia/20/99 A/H3N2 similar to A/Brisbane/10/07 For 2008: similar to A/H1N1/Brisbane/59/07	Influenza B (9.7%) Type B similar to B/Malaysia/ 2506/07 and similar to B/Florida/ 4/06-like	Parainfluenza: 3.2%, adenovirus: 1.8%, RSV: 0.6%, coinfections: 20.7%
2007 - 2008^ (^ ^7^	12,395	39% (IFA)	Influenza A 5% A/H1N1 similar to Brisbane/59/2007	Influenza B 2%, B/Hong Kong/330/2001, B/Shanghai/361/2002	Adenovirus: 15%, RSV: 7%, Parainfluenza: 2.4%, Parainfluenza: 1.3%, Parainfluenza: 3.3%
2009 ^8^ ^,^ ^13^	9291 for rRT-PCR	19% (1771)	85% Influenza A(H1N1)v, 15% Seasonal Influenza	∼ B/Florida	??
2010^ (^ ^13^ ^,^ ^14^	2618 for rRT-PCR	23.6 % (618)	A (H1N1) pdm09: 62% A/H3N2: 18%	20% Influenza B: B/Victoria, B/Yamagata and not typifiable	Increase in RSV circulation in childhood pneumonia
2013 ^15^ ^,^ ^16^	11,832 for rRT-PCR and DFA	12.86% (1522) Positives, 179 IFA 441 rRT-PCR 26 cultures	AH1N1, 29.3% from Lima and 12.8% from Piura 15.24% (232) Influenza AH3N2	21.22% (323) influenza B cases	RSV: 3.81%
2014 ^(INS Bulletins 2014)^	6678 for rRT-PCR to influenza 2792 for IFA	10.37% (693)	A/H1N1: 189 (27.27%) A/H3N2: 279 (40.25%)	Influenza B: 225 (32.46 %)	RSV: 15.79%
2015 ^(INS Bulletins 2015)^	4283 for IFA, DFA and rRT-PCR 1754 for ORV	10.78% (462)	A/H1N1: 124 (26.83%) A/H3N2: 287 (62.12%)	Influenza B: 51 (11.03%)	RSV: 14.36%
2016 ^(INS Bulletins 2016)^	4754 for rRT-PCR	33.6% (1595)	Flu A: 45.8 % (731) A/ H1N1pdm09: 88.5% (647) A/H3N2: 11.5% (84)	Flu B: 25.2% (402) Influenza B Victoria 5% Influenza B Yamagata 15%	RSV: 20.1% (321) Metapneumovirus: (2.2%), Rhinovirus: (2.1%), Adenovirus: (1.3%), Parainfluenza: 3 (1.5%), Parainfluenza: 1 (1.0%) and Parainfluenza: 2 (0.9%).
2017^ (INS Bulletins 2017)^	1466 for rRT-PCR	29.9% (438)	A/H1N1: 1.37% A/H3N2: 214 (48.85%)	Non-subtyped Influenza B: 14% Influenza B/Victoria 11.2% B/Yamagata 5.6%	Rhinovirus: 36% Metapneumovirus: 26% RSV: 17%, Adenovirus: 4.5% Parainfluenza: 15%
2018 ^(NETLAB v.01)^	1389 for rRT-PCR	23.6% (328)	A/H1N1pdm09: 175 (53.35%) A/H3N2: 63 (19.20%)	B/Yamagata: 3.09 % B/Victoria: 0.86 %	RSV: 58% Parainfluenza: 22% Metapneumovirus: 15% Adenovirus: 4%, rhinovirus: 1%
2019 ^(NETLAB v.02)^	4244 for rRT-PCR 2699 for DFA	25.94% (1101)	A/H1N1pdm09: 74 (6.72%) A/H3N2: 322 (29.24%)	B/Yamagata, 74 (6.7%) 45 (4.08%) B/Victoria and 9 (0.81%) B not-subtyped	RSV: 399 (36.23%) Rhinovirus: 16% Metapneumovirus; 8%
2020 ^(NETLAB v.02)^	1,516,208 for SARS-CoV-2 rRT-PCR, Influenza/ORV multiplex For DFA (only until March 2020: 2156)	16% (235,531) 2% (37) Influenza 28% (603) ORV	A/H1N1pdm09: 05 (13.5%) A/H3N2: 20 (54%)	B/Victoria 08 (21.6%) B/Yamagata 03 (8%) Flu B 01 (2.7%)	SARS-COV-2 Parainfluenza: 3 7 (1.2%) Rhinovirus: 164 (27.2%) Metapneumovirus: 344 (57%) Adenovirus: 11 (1.8%)

IFA: indirect immunofluorescence. DFA: direct immunofluorescence. Flu: influenza. RSV: respiratory syncytial virus. rRT-PCR: real-time reverse transcription-polymerase chain reaction. SARS-CoV-2: severe acute respiratory syndrome coronavirus type 2.

A/H3N2/Panama/2007/99: influenza type A/subtype H3N2/Location of finding/Finding number/Year of finding

A/H1N1pdm09: influenza type A/subtype H1N1 pandemic strain of 2009.

B/Sichuan/ 379 / 99: type B/Lineage with name of place of finding/year of finding

A (H1N1) v: Influenza virus type A, subtype (H1N1)

Surveillance of SARI was strengthened by selecting sentinel units according to the influx of patients, geographic location and residence in the area. In 2005, there were 15 units and the following year, due to dissemination and motivation, that number increased to 50. Training was improved in laboratories collaborating with the RNL, in order to obtain, according to case definition, nasal and pharyngeal swab samples in virus transport media (VTM), and to send aliquots, in cold chain, to the INS ^5^. Since 2001, several RRLs with *ad hoc* personnel and equipment have performed influenza and ORV diagnosis by indirect or direct immunofluorescence (IFA or DFA) with commercial kits ^6^.

The first influenza A (H1N1) pdm09 pandemic of the 21st century prompted the strengthening of surveillance for the detection, notification and control of the virus ^7^. Three years after the pandemic, new strains of influenza were detected that continued to circulate as seasonal viruses, severely affecting infants and the elderly ^8^ (Table 1).

The health regulations that supported surveillance of influenza virus and ORV were approved in 2012 and established basic guidelines for dealing with SARI in Peru ^9^. The guidelines were updated in 2014, in response to the WHO recommendation to develop anti-pandemic plans due to evidence of outbreaks of influenza A H7N9 in China and the MERS CoV-1 respiratory syndrome coronavirus in the Middle East ^10^.

In 2016, when DFA kits with monoclonal antibodies for metapneumovirus and rhinovirus were introduced, they were included as part of the differential diagnosis process of ORV, and these viruses were detected in significant percentages during 2017 (Table 1). The influenza laboratory surveillance protocol was redesigned in 2018 and 2019 to strengthen the capacity for data collection, processing, data analysis and quality control.

## PROCESS

### Development of molecular tests for the diagnosis of respiratory viruses.

The sequence of events related to molecular diagnosis during surveillance (Figure 1) began when the LRNVR managed to incorporate molecular tests to identify influenza and ORV, coordinating not only with the CDC in Atlanta, but also with laboratories in France, Canada, and the Tropical Diseases Research Institute of the U.S. Navy’s Naval Medical Detachment (NAMRID), based in Lima ^6^. A few years later, trials for the standardization of the conventional polymerase chain reaction (PCR) method to detect influenza virus were conducted at LRNVR.

The reverse transcription PCR (RT-PCR) technique was standardized in 2006 for surveillance and early detection of the A/H5N1 influenza virus, in response to an alert regarding the presence of human cases of avian influenza in Asia, Europe and Africa ^11^. A 2007 technical document included this molecular test as part of the laboratory diagnosis of respiratory viruses in Peru ^12^.

In 2009, during the H1N1pdm09 pandemic, real-time RT-PCR (rRT-PCR) molecular diagnosis was implemented according to CDC protocols, based on nasal and/or pharyngeal samples from suspected cases, which were submitted to the INS ^13^ ^,^ ^14^. The influenza virus pandemic revealed specific deficiencies and functional weaknesses in surveillance, with absence of historical data ^15^.

In 2010, diagnostic activity increased (Table 1) and positive cases were detected, not only of the pandemic strain, but also of seasonal influenza and RSV ^16^. Two years later, a situational analysis of national influenza surveillance was carried out ^17^.

In 2012, the RRL of Cusco was identified as a laboratory with the capacity to perform RT-PCR ^18^, a method transferred by the INS.

In 2013, those responsible for surveillance highlighted achievements in determining viral etiology and strengthening the timely diagnosis of SARI by rRT-PCR; however, they detected deficiencies in the filling out of clinical-epidemiological records by sentinel units, which made it difficult to differentiate cases of FS and SARI, related to etiological findings ^19^.

In 2014, the LRNVR conducted rRT-PCR validation assays to identify RSV and its subtypes, a pathogen in children and infants with SARI.

The Multiplex RT-PCR method to identify influenza A, B viruses and subtypes was validated in 2017 ^20^, and subsequently standardized to include molecular detection of RSV and rhinovirus, among other RSVs, depending on the availability of primers and probes, mainly by donations from the CDC.

In 2018, the RRLs of Tumbes and Piura were trained in rRT-PCR for the diagnosis of respiratory viruses, within the TT of methods performed by INS.

### Execution of molecular sequencing and genomic analysis studies

In the first 10 years of surveillance, positive samples and influenza isolates were sent to the CDC for molecular characterization. In 2010, during the pandemic, the INS acquired equipment for sequencing at the Biomedicine center, with advice from specialists from the Biotechnology and Molecular Biology Laboratory (LBBM) of the CNSP. In 2013, the complete genome analysis of the influenza A(H1N1)pdm09 virus began ^21^.

In 2015 and 2016, when characterizing influenza A(H1N1)pdm09 genotypes in clinical samples, massive whole genome sequencing or Next-Generation Sequencing (NGS) proved to be more sensitive and efficient than the High Resolution Melting (HRM) technique, which analyzed only two genes ^22^.

Subsequently, it was possible to characterize, through massive sequencing, the hemagglutinin and neuraminidase genes in influenza virus isolates, achieving a global and timely vision of the circulating strains in Peruvian territory and identifying genetic variations related to antiviral resistance. In 2019, by amplifying fragments of isolates and analyzing the complete genome of the influenza virus by NGS, information was obtained on circulating viruses and mutations without implication of the effect on drugs.

These previous experiences were used to sequence SARS-CoV-2 isolates obtained from COVID-19 cases in Peru ^23^; information was also obtained on local transmission in the early phase of the pandemic ^24^. Although initially considered costly and complex, sequencing is now indispensable for monitoring circulating variants of the pandemic virus.

### Aspects of quality control and information flow in the reporting of results

The rRT-PCR method for influenza virus detection was officialized in 2018, according to Peruvian technical standard (NTP) 15189-2014 ^25^. All methods used in surveillance are subject to quality management; the LRNVR takes part in external quality control programs of rRT-PCR for influenza, which are authorized by WHO and organized by the CDC in Atlanta and the Center for Health Protection in Hong Kong, sending typed and subtyped isolates. It is currently certified by the National Quality Institute (INACAL), in coordination with the CNSP Quality Management Unit. The RRLs participate in the Annual External Quality Assessment Program scheduled by the INS.

In 2007, the NETLAB computer system was created at INS to enter and communicate laboratory results in a timely manner, both at the central and regional levels. Since 2019, a new version has been available with innovative technology for the collection, analysis, storage and management of data in real time; it uses the unit of measurement: “diagnosed person”, improving the quality of information and its usefulness for surveillance. Results continue to be reported weekly to WHO through FluNet.

### Laboratory Surveillance Response during the COVID-19 pandemic

In 2020, once the state of emergency was declared in Peru due to the COVID-19 outbreak, the diagnostic confirmation algorithm for SARS-CoV-2 was defined; the LRNVR undertook the molecular diagnosis of all probable cases at the national level, using rRT-PCR, according to the reference protocol recommended by WHO ^26^. At the same time, an in-house real-time rRT-PCR test was developed and validated using the specific RdRp gene and endogenous GAPDH control, in order to shorten the time required to issue results and optimize resources ^27^.

From February to March 2020, the average diagnostic capacity of the LRNVR was 500 tests per day. Given the exponential increase in the demand for testing due to the pandemic, other Biomedicine LRNs with capacity in molecular procedures, such as those for mycobacteria, sexually transmitted viruses HIV/AIDS and viral metaxenics, were called upon for support. Pre-analytical, analytical and post-analytical activities carried out by working groups on 24-hour shifts, seven days a week reached a daily processing of 1725 samples. On June 16, 2020, with the inauguration of the new molecular diagnostic laboratory exclusively for SARS-CoV-2, the response capacity increased to 5,000 tests per day, on average. In addition, automated and semi-automated platforms for SARS-CoV-2 diagnosis by rRT-PCR from nasal and pharyngeal swabs were implemented, including Qiagen, GeneXpert and Cobas®. All results were recorded in the NETLAB.v.2.0 information system.

In 2021, 20,414,072 human samples were processed nationwide; 2,239,421 were confirmatory for COVID-19 infection. Of the 5,405,940 molecular tests in the country, 30.6% (1,653,795) were performed at the INS, almost one million more than those processed in 2020, at a rate of approximately 8,000 tests per day, in its five laboratories: Chorrillos, Lima, Loreto and three mobile laboratories. So far in 2022, more than 10,000 tests have been processed daily.

From September to December 2020, more than 10,000 isothermal RT-LAMP tests were performed for the diagnosis of COVID-19, an alternative molecular method with few equipment requirements, fast results and lower costs, very useful for the first level of care, that, after validation ^28^, was implemented in several sites in Lima, Callao and Pasco.

As a result of the spread of COVID-19 in several regions of the country, the INS implemented molecular diagnosis initially in nine regions, and has continued to strengthen the RNL to decentralize diagnosis. Of 1,514,718 molecular tests processed in 2020 for COVID-19 diagnosis, 49% were carried out by the RNL; similarly, the 235,531 SARS-CoV-2 positive cases (36.6% of the national total processed) were detected by the RNL. To date, there are 28 RRLs authorized to perform molecular diagnosis of COVID-19. After evaluation, laboratories of various levels were progressively approved, bringing the total number of authorized laboratories to 126. It is necessary to achieve a high diagnostic capacity because more molecular tests are known to ensure good control of the pandemic ^29^.

Genomic data for circulating pathogens in several Latin American countries were scarce or non-existent; with the advent of SARS-CoV-2, the situation changed radically, but the available information is still insufficient ^30^.

## CONCLUSIONS

In the current COVID-19 pandemic, the implementation of molecular testing and the participation of laboratories specialized in identifying new viruses, such as SARS-CoV-2, have been essential to control the impact on public health. At the beginning of the pandemic, the LRNVR was the only laboratory in the country for the molecular detection of new respiratory viruses as part of the laboratory surveillance of influenza and ORV, with an initial processing capacity of 500 molecular tests per day, which progressively increased to more than 10,000 molecular tests per day at present.

In 2019, there were three RRLs trained by TT to perform molecular tests for the diagnosis of respiratory viruses within the surveillance of Influenza and ORV. Currently, due to the need to respond to the pandemic, 28 RRLs have incorporated molecular methods to their diagnostic capabilities, through accelerated TT processes. In the last decade, during the influenza A(H1N1) pdm09 pandemic, the INS acquired equipment and improved its capabilities to perform genomic sequencing. Currently, SARS-CoV-2 surveillance routinely involves genomic analysis by molecular sequencing for changes in pandemic virus variants.

The strengthening and innovation of biotechnological advances for laboratory-based surveillance, with own resources and external support, as well as the increase in NLR capacities, enabled the generation of timely and quality information for diagnostic confirmation during the current pandemic. It is necessary to consolidate this system in a collaborative and coordinated manner, taking into account the diverse regional contexts and the complex inter-institutional interactions. The infrastructure, resources and experience accumulated over the last twenty-two years to develop molecular diagnostic methods in Peru, as part of the laboratory surveillance of influenza and ORV, has served as the basis for the current laboratory monitoring of SARS-CoV-2.
