# Supplementary material for: Laboratory platform for monitoring SARS-CoV-2 based on surveillance of influenza and other respiratory viruses in Peru
Source: Rev Peru Med Exp Salud Publica. 2022 Mar 31;39(1):104–10. doi: 10.17843/rpmesp.2022.391.8380 (PMC11397776; doi:10.17843/rpmesp.2022.391.8380)
Supplement: Translation

# Translation: Plataforma laboratorial para monitorear el SARS-CoV-2 basada en la vigilancia de influenza y otros virus respiratorios en Perú

**Authors:** Rosa Palacios-Salvatierra, Maribel Huaringa-Núñez, Priscila Lope-Pari, Johanna Balbuena-Torres, Nancy Rojas-Serrano

**Keywords:** Vigilancia en Salud Publica, Vigilancia Sanitaria, Servicios de Vigilancia Epidemiológica, Monitoreo Epidemiológico, Virus de la Influenza A, Virus de la Influenza B, Técnicas de diagnóstico molecular, Prueba de COVID-19, Servicios Laboratoriales de Salud Pública, Sistemas Nacionales de Salud

## Abstract

En el Perú, la pandemia de la COVID-19 ha evidenciado la utilidad de tener un sistema de vigilancia laboratorial estructurado y en funcionamiento desde hace 22 años, basado en la vigilancia de influenza; inicialmente en modalidad de unidades centinela, y después fortaleciéndose e innovándose, con recursos propios y con apoyo externo, para generar información de calidad. Se han implementado avances biotecnológicos para la confirmación diagnóstica e incrementado las capacidades de la red nacional de laboratorios, manteniendo la eficiencia, considerando las diversas y complejas realidades de los niveles regionales, y superando dificultades de comunicación y articulación entre instituciones. Resulta necesario consolidar este sistema, con trabajo colaborativo y coordinado entre sus componentes, impulsando su eficacia y oportunidad y promoviendo la vigilancia genómica de nuevos virus y variantes, como actualmente ocurre con el SARS-CoV-2.

## INTRODUCCIÓN

La rápida diseminación del coronavirus causante del síndrome respiratorio agudo severo (SARS-CoV-2), su alta transmisibilidad, morbilidad, mortalidad, e impacto socioeconómico en todo el mundo^ (^ ^1^, impulsó el establecimiento de sistemas de vigilancia con monitoreo constante, mediante pruebas moleculares, para confirmar casos sospechosos.

En el Perú, al inicio de la pandemia, este reto fue afrontado por el Laboratorio de Referencia Nacional de Virus Respiratorios (LRNVR) del Centro Nacional de Salud Pública (CNSP) del Instituto Nacional de Salud (INS), en la sede de Biomedicina en Chorrillos, Lima. El LRNVR es un centro colaborador de la Organización Mundial de la Salud (OMS), para la vigilancia epidemiológica laboratorial de influenza y otros virus respiratorios (OVR) que había iniciado la transferencia tecnológica (TT) de métodos hacia determinados laboratorios de referencia regional (LRR) de la Red Nacional de Laboratorios (RNL).

El presente artículo recopila información sobre la implementación de esta vigilancia laboratorial durante los últimos 22 años, con énfasis en el desarrollo de pruebas moleculares, revisando para ello fuentes primarias y secundarias como informes, reportes, publicaciones, entre otros, estableciendo una secuencia de procesos en el tiempo (Figura 1).

**Figura 1:** Línea de tiempo de la implementación de pruebas moleculares para la vigilancia de virus respiratorios en el Perú, 1999-2021.

El objetivo de esta publicación fue evidenciar que, ante la pandemia de la COVID-19, en el Perú existía un sistema de vigilancia laboratorial para detectar nuevos virus respiratorios, nuevas cepas y linajes, con pruebas moleculares y secuenciamiento genómico, realizados en el INS y en algunos LRR, por especialistas entrenados. Estas capacidades se generaron durante la vigilancia del virus de la influenza y ha servido de base ante los retos del actual virus pandémico. 

## ANTECEDENTES

La vigilancia de virus respiratorios basada en laboratorio en el Perú fue establecida para detectar, identificar y caracterizar la etiología, actividad y circulación del virus de la influenza y OVR durante casos de infecciones respiratorias agudas graves (IRAG) y enfermedad tipo influenza/síndrome gripal (ETI/SG) con la participación de los centros centinela a nivel nacional. Se priorizó el diagnóstico diferencial del virus de la influenza con OVR, inicialmente adenovirus, parainfluenza 1, 2 y 3, virus sincicial respiratorio (VSR), y posteriormente rinovirus y metapneumovirus humano. Además de su notificación oportuna, se contribuyó a formular la vacuna de influenza, detectar el surgimiento de variantes u otros patógenos emergentes con potencial epidémico o pandémico y monitorear técnicamente a los LRR comprometidos con esta vigilancia.

El LRNVR participa en la vigilancia de influenza y OVR desde 1999 ^2^ como centro colaborador de la OMS, remitiendo información a través del sistema informático FluNet, al Centro de Control de Enfermedades Transmisibles (CDC) de Atlanta en Estados Unidos (EE. UU), y enviando muestras positivas tipificadas y aislamientos virales seleccionados para su caracterización antigénica y estudio genético molecular ^3^. Así, pudo confirmarse la etiología viral del síndrome respiratorio febril agudo y el ingreso al Perú de nuevas cepas de influenza (Tabla 1), en una época en que los sistemas de vigilancia de los países en desarrollo no exhibían suficiente capacidad para ello ^4^.

**Tabla 1:** Resultados de la vigilancia laboratorial de influenza y otros virus respiratorios en Perú (2000-2020).

Año	N.° Muestras	% Positivos	Influenza A: tipos y subtipos	Influenza B: linajes	OVR
2000 ^2^	600	67,6% (406) por IFI y por aislamiento	A(H3N2) 28,3%, similar a: A/H3N2/Panamá/2007/99	Flu B: 29,5% B/Beijing Yamanashi/166/98 (inicios 2000), luego B/Sichuan/379/99	VSR 2,6%, parainfluenza 5,2%, adenovirus 2,0%
2001 ^2^ ^,^ ^5^ ^,^ ^6^	1103	85,67% (944) por IFI	A(H3N2) 55 - 60%, similar a: A/H3N2/Panamá/2007/99	Flu B: 22% 16,50%: B/Beijing Yamanashi/166/98	VSR 2,6-3%, parainfluenza 8,07-11%, adenovirus 3 - 4%
2002 ^2^ ^,^ ^5^ ^,^ ^6^	1327	74,98% (995) por IFI	Influenza A (H3N2/2007/99) 13,94%	Influenza B/ Sichuan/379/99 10,12%	Parainfluenza 21,55%, VSR 13,33%, adenovirus 12%
2004 ^4^	2375	60,8% (1444)	Influenza A 31,65 % (457)	Influenza B: 10% (144)	Adenovirus: 18 % (261) parainfluenza 10,2% (147) VSR: 30,12% (435)
2005^4^	87 ( 2-15 enero)	56%	Influenza A/H1 38% Influenza /H3 32%	Influenza B 2,9% Influenza B: 1,9%	Adenovirus: 1ra semana: 35%, 2da semana: 3,77% VSR: 1ra semana: 5,8%, 2da semana: 1,9%
2006-2008 ^11^ ^,^ ^26^	6835	Vigilancia Enero-febrero 2006: 37,23% de positivos x IFD	Influenza A 25% Para 2006: similar a A/H1N1/Solomon Island/03/06 y similar a A/H1N1/New Caledonia/20/99 A/H3N2 similar a A/Brisbane/10/07 Para 2008: similar a A/H1N1/Brisbane/59/07	Influenza B (9,7%) Tipo B similar a B/Malaysia/2506/07 y similar a B/Florida/ 4/06-like	Parainfluenza 3,2%, adenovirus 1.8%, VSR 0,6%, coinfecciones 20,7%
2007 - 2008 ^7^	12 395	39% (IFI)	Influenza A 5% A/H1N1 similar a Brisbane/59/2007	Influenza B 2%, B/Hong Kong/330/2001, B/Shanghai/361/2002	Adenovirus 15%, VSR 7%, Parainfluenza 2,4%, Parainfluenza 1,3%, Parainfluenza 3,3%
2009 ^8^ ^,^ ^13^	9291 para rRT-PCR	19% (1771)	85% Influenza A(H1N1)v, 15% Influenza estacional	∼ B/Florida	??
2010 ^13^ ^,^ ^14^	2618 para rRT-PCR	23,6 % (618)	A (H1N1) pdm09: 62% A/H3N2: 18%	20% Influenza B: B/Victoria, B/Yamagata y no tipificables	Incremento en circulación de VSR en neumonía infantil
2013 ^15^ ^,^ ^16^	11 832 para rRT-PCR e IFD	Positivas 12,86% (1522), 179 IFD 441 rRT-PCR 26 cultivos	AH1N1, 29,3% de Lima y 12,8% de Piura 15,24% (232) Influenza AH3N2	21,22% (323) casos de influenza B	VSR 3.81%
2014 ^(Boletines INS 2014)^	6678 para rRT-PCR a Influenza 2,792 para IFD	10,37% (693)	A/H1N1: 189 (27,27%) A/H3N2: 279 (40,25%)	Influenza B: 225 (32,46 %)	VSR 15.79%
2015 ^(Boletines INS 2015)^	4283 para IFI, IFD y rRT-PCR 1754 para OVRs	10,78% (462)	A/H1N1: 124 (26,83%) A/H3N2: 287 (62,12%)	Influenza B: 51 (11,03 %)	VSR 14.36%
2016 ^(Boletines INS 2016)^	4754 para rRT-PCR	33,6% (1,595)	Flu A: 45.8 % (731) A/ H1N1pdm09: 88,5% (647) A/H3N2: 11,5% (84)	Flu B: 25,2 % (402) Influenza B Victoria 5% Influenza B Yamagata 15%	VSR: 20,1% (321) Metapneumovirus (2,2%), Rinovirus (2,1%), Adenovirus (1,3%), Parainfluenza 3 (1,5%), Parainfluenza 1 (1,0%) y Parainfluenza 2 (0,9%).
2017 ^(Boletines INS 2017)^	1466 para rRT-PCR	29,9% (438)	A/H1N1: 1,37% A/H3N2: 214 (48,85%)	Influenza B no subtipificado: 14% Influenza B/Victoria 11,2% B/Yamagata 5,6 %	Rinovirus: 36% Metapneumovirus: 26% VSR: 17%, Adenovirus 4,5% Parainfluenza: 15%
2018 ^(NETLAB v.01)^	1389 para rRT-PCR	23,6% (328)	A/H1N1pdm09: 175 (53,35%) A/H3N2: 63 (19,20%)	B/Yamagata: 3,09 % B/Victoria: 0,86 %	VSR 58% Parainfluenza 22% Metapneumovirus 15% Adenovirus 4%, rinovirus 1%
2019 ^(NETLAB v.02)^	4244 para rRT-PCR 2699 para IFD	25,94 % (1,101)	A/H1N1pdm09: 74 (6,72%) A/H3N2: 322 (29,24%)	B/Yamagata, 74 (6,7%) (45 (4,08%) B/Victoria y 9 (0,81%) B no subtipificada	VSR: 399 (36,23%) Rinovirus: 16% Metapneumovirus; 8%
2020 ^(NETLAB v.02)^	1 516 208 para rRT-PCR SARS-CoV-2, Multiplex de Influenza/OVR Para IFD (solo hasta marzo 2020: 2156)	16% (235,531) 2% (37) Influenza 28% (603) OVR	A/H1N1pdm09: 05 (13,5%) A/H3N2: 20 (54%)	B/Victoria 08 (21,6%) B/Yamagata 03 (8%) Flu B 01 (2,7%)	SARS-COV-2 Parainfluenza 3 7 (1,2%) Rinovirus 164 (27,2%) Metapneumovirus 344 (57%) Adenovirus 11 (1,8%)

IFI: inmunofluorescencia indirecta. IFD: inmunofluorescencia directa. Flu: influenza. VSR: virus sincicial respiratorio. OVR: otros virus respiratorios. rRT-PCR: prueba de reacción en cadena de la polimerasa con transcripción reversa en tiempo real. SARS-CoV-2: coronavirus tipo 2 causante del síndrome respiratorio agudo severo.

A/H3N2/Panamá/2007/99: influenza tipo A/subtipo H3N2/Lugar de hallazgo/Numero de hallazgo/año de hallazgo

A/H1N1pdm09: influenza tipo A/subtipo H1N1 cepa pandémica del año 2009.

B/Sichuan/ 379 / 99: tipo B/Linaje con el nombre del lugar del hallazgo/año del hallazgo

A (H1N1) v: virus Influenza tipo A, subtipo (H1N1)

La vigilancia de las IRAG fue fortalecida seleccionando unidades centinela según afluencia de pacientes, por ubicación geográfica y residencia en el área. En el año 2005 existían 15 unidades y al año siguiente, por difusión y motivación, aumentaron a 50. Fue intensificada la capacitación a laboratorios colaboradores de la RNL, a fin de obtener, según definición de caso, muestras de hisopados nasal y faríngeo en medios de transporte para virus (MTV), y remitir alícuotas, en cadena de frio, hacia el INS ^5^. Desde el año 2001 varios LRR con personal y equipo *ad hoc* han realizado el diagnóstico de influenza y OVR por inmunofluorescencia indirecta o directa (IFI o IFD) con kits comerciales ^6^.

La primera pandemia del siglo XXI por influenza A (H1N1) pdm09, motivó a fortalecer la vigilancia para la detección, notificación y control del virus ^7^. Luego de tres años pospandemia, se detectaron cepas nuevas de influenza que continuaban circulando como virus estacionales, afectando severamente a infantes y ancianos ^8^ (Tabla 1). 

La directiva sanitaria que sustentó la vigilancia del virus de la influenza y OVR, fue aprobada en el año 2012, en ella se establecieron lineamientos básicos para enfrentar a las IRAG en el Perú ^9^. Las directivas fueron actualizadas el año 2014, ante la recomendación de la OMS para elaborar planes antipandémicos por evidencia de brotes de influenza A H7N9 en China y de coronavirus del síndrome respiratorio en el Medio Oriente MERS CoV-1^ (^ ^10^.

En el 2016, al incorporarse kits de IFD con anticuerpos monoclonales para metapneumovirus y rinovirus, estos fueron incluidos en el diagnóstico diferencial de OVR, logrando detectar estos virus en porcentajes significativos en el 2017 (Tabla 1). El protocolo de vigilancia laboratorial de influenza fue reformulado en los años 2018 y 2019 para reforzar la capacidad en colecta de información, procesamiento, análisis de datos y control de calidad.

## EL PROCESO

### Desarrollo de las pruebas moleculares para diagnóstico de virus respiratorios

La secuencia de hechos relacionados al diagnóstico molecular durante la vigilancia (Figura 1), se inició, cuando el LRNVR gestiono incorporar pruebas moleculares para identificar influenza y OVR, coordinando no solo con el CDC de Atlanta, sino con laboratorios de Francia, Canadá, y con el Instituto de Investigación de Enfermedades Tropicales del Destacamento Medico Naval de la Marina de EE. UU. (NAMRID), con sede en Lima ^6^. Pocos años después, en el LRNVR se realizaron ensayos para la estandarización del método de reacción en cadena de la polimerasa (PCR) convencional para detectar el virus de la influenza.

La técnica de PCR con transcripción reversa (RT-PCR) fue estandarizada el año 2006 para vigilancia y detección temprana del virus de influenza A/H5N1, a propósito de una alerta ante la presencia de casos humanos de esta gripe aviar en Asia, Europa y África, ^11^. Un documento técnico del año 2007 incluyó esta prueba molecular en el diagnóstico de laboratorio de virus respiratorios en el Perú ^12^. 

En el 2009, durante la pandemia por el virus H1N1pdm09, fue implementado el diagnóstico molecular de RT-PCR en tiempo real (rRT-PCR) según protocolos del CDC, a partir de muestras nasales y/o faríngeas de casos sospechosos, que eran remitidas al INS ^13^ ^,^ ^14^. La pandemia por el virus de la influenza evidenció deficiencias específicas y debilidades funcionales en la vigilancia, con ausencia de datos históricos^ (^ ^15^.

En el 2010 hubo abundante actividad diagnóstica (Tabla 1) y se detectaron casos positivos, no solo de la cepa pandémica, sino también de la influenza estacional y OVR ^16^. Dos años después se realizó un análisis situacional de la vigilancia nacional de influenza ^17^. 

En el 2012, el LRR de Cusco era identificado como laboratorio con capacidad para realizar RT-PCR ^18^, método transferido por el INS.

En el 2013, los responsables de la vigilancia destacaron los logros en la determinación de etiología viral y fortalecimiento del diagnóstico oportuno de IRAG por rRT-PCR; sin embargo, detectaron deficiencias en el llenado de fichas clínico-epidemiológicas por las unidades centinela, lo que dificultó diferenciar los casos de SG e IRAG, relacionados a los hallazgos etiológicos ^19^.

En el 2014, el LRNVR realizo ensayos de validación del rRT-PCR para identificar al VSR y sus subtipos, un patógeno de niños e infantes con IRAG. 

El método RT-PCR Múltiplex para identificar virus de influenza A, B y subtipos, fue validado en el 2017 ^20^, y posteriormente estandarizado para incluir la detección molecular del VSR y rinovirus, entre otros OVR, según disponibilidad de iniciadores y sondas, por donaciones, principalmente del CDC.

En el 2018, los LRR de Tumbes y Piura fueron capacitados en rRT-PCR para el diagnóstico de virus respiratorios, dentro de la TT de métodos que realiza el INS.

### Ejecución de secuenciamientos moleculares y estudios de análisis genómico

En los primeros 10 años de la vigilancia, las muestras positivas y aislamientos de influenza se remitían al CDC para su caracterización molecular. En el 2010, durante la pandemia, el INS adquirió equipos para secuenciamiento en la sede de Biomedicina, con asesoría de especialistas del Laboratorio de Biotecnología y Biología Molecular (LBBM) del CNSP. El 2013 comenzó el análisis completo del genoma del virus influenza A(H1N1)pdm09 ^21^. 

En el 2015 y 2016, al caracterizar los genotipos de influenza A(H1N1)pdm09 en muestras clínicas, el secuenciamiento masivo de genoma completo-Next Generation Sequence (NGS) resultó más sensible y eficiente que la técnica High Resolution Melting (HRM), que analizaba solo dos genes ^22^.

Posteriormente, fue posible caracterizar, mediante secuenciamiento masivo, los genes hemaglutinina y neuraminidasa en aislados del virus de la influenza, logrando una visión global y oportuna de las cepas circulantes en territorio peruano e identificando variaciones genéticas relacionadas a la resistencia para antivirales. En el 2019, al amplificar fragmentos de aislamientos y analizar el genoma completo del virus de la influenza por NGS, se obtuvo información sobre virus circulantes y mutaciones sin implicancia de efecto sobre fármacos. 

Estas experiencias previas sirvieron para lograr secuenciar aislamientos del SARS-CoV-2 obtenidos de casos de COVID-19 en el Perú ^23^; asimismo se obtuvo información sobre transmisión local en fase pandémica temprana ^24^. Si bien en un principio eran considerados costosos y complejos, actualmente los secuenciamientos son indispensables para monitorear variantes circulantes del virus pandémico.

### Aspectos del control de calidad y flujo en información de resultados

El método rRT-PCR para la detección del virus de la influenza fue oficializado en el 2018, según la norma técnica peruana (NTP) 15189-2014^ (^ ^25^. Todos los métodos empleados en la vigilancia están sujetos a gestión de calidad; el LRNVR participa en programas de control de calidad externo del rRT-PCR para influenza, autorizados por la OMS y organizados por el CDC de Atlanta y el Center for Health Protection de Hong Kong, enviando aislamientos tipificados y subtipificados. Actualmente, cuenta con acreditación por el Instituto Nacional de Calidad (INACAL), en coordinación con la Unidad de Gestión de la Calidad del CNSP. Los LRR participan en el Programa Anual de Evaluación Externa de la Calidad programado por el INS.

En el 2007 fue creado el sistema informático NETLAB en el INS, para ingresar y comunicar oportunamente los resultados de laboratorio, tanto a nivel central y regional. Desde el 2019 existe una nueva versión con tecnología innovadora para colecta, análisis, almacenamiento y gestión de datos en tiempo real, con unidad de medida de «persona diagnosticada», mejorando la calidad de información y su utilidad para la vigilancia. Semanalmente continúan reportándose resultados a la OMS mediante FluNet. 

### Respuesta de la vigilancia laboratorial durante la pandemia de la COVID-19

En el 2020, una vez declarado el estado de emergencia por el brote de la COVID-19 en el país, fue definido el algoritmo de confirmación diagnóstica para SARS-CoV-2; el LRNVR asumió el diagnóstico molecular de todos los casos probables a nivel nacional, mediante rRT-PCR, según protocolo referencial recomendado por la OMS ^26^. Paralelamente, fue desarrollada y validada una prueba rRT-PCR en tiempo real *in house* usando gen especifico RdRp y control endógeno GAPDH, a fin de acortar tiempos en la emisión de resultados y optimizar recursos ^27^. 

Desde febrero a marzo de 2020, la capacidad diagnóstica promedio del LRNVR fue de 500 pruebas diarias. Ante el incremento exponencial en la demanda de pruebas por la pandemia, se recurrió al apoyo de otros LRN de Biomedicina, con capacidad en procedimientos moleculares, como los de micobacterias, virus de transmisión sexual VIH/SIDA y metaxénicas virales. Las actividades preanalíticas, analíticas y posanalíticas realizadas en jornadas de 24 h durante siete días a la semana, en grupos de trabajo, alcanzaron a procesar 1725 muestras diarias. El 16 de junio de 2020, al inaugurarse el nuevo laboratorio de diagnóstico molecular exclusivo para SARS-CoV-2 la capacidad de respuesta aumentó a 5000 pruebas diarias, en promedio. Además, fueron implementadas plataformas automatizadas y semiautomatizadas para el diagnóstico del SARS-CoV-2 mediante rRT-PCR a partir de hisopados nasales y faríngeos, entre ellas Qiagen, GeneXpert y Cobas ®. Todos los resultados fueron registrados en el sistema de información NETLAB.v.2.0. 

En el 2021 se procesaron 20 414 072 muestras humanas a nivel nacional; 2 239 421 fueron confirmatorias de infección por la COVID-19. De los 5 405 940 de pruebas moleculares en el país, en el INS se realizaron el 30,6% (1 653 795), casi un millón más de las procesadas en el 2020, a razón de aproximadamente 8000 pruebas diarias, en sus cinco laboratorios: Chorrillos, Lima, Loreto y tres laboratorios móviles. En lo que va del 2022, se han procesado más de 10 000 pruebas diarias.

De septiembre a diciembre de 2020, se realizaron más de 10 000 pruebas isotérmicas RT-LAMP para diagnóstico de la COVID-19, método molecular alternativo con pocos requerimientos de equipos, rápidos resultados y costos menores, muy útil para el primer nivel de atención, que, previa validación ^28^, fue implementado en varias sedes de Lima, Callao y Pasco.

A consecuencia de la presentación de la COVID-19 en varias regiones del país, el INS implementó el diagnóstico molecular, en un principio en nueve regiones, y ha continuado fortaleciendo la RNL y descentralizando el diagnostico. De 1 514 718 pruebas moleculares procesadas en el 2020 para el diagnóstico de COVID-19, el 49% estuvo a cargo de la RNL; de igual forma, los 235 531 casos positivos a SARS-CoV-2 (36,6% del total nacional procesado) han procedido de LRR. A la fecha, existen 28 LRR autorizados para realizar diagnóstico molecular de la COVID-19. Previa calificación, progresivamente se autorizaron laboratorios de diversas instancias por lo que actualmente son 126 los autorizados. Es necesario alcanzar altas capacidades diagnósticas porque se sabe que la ejecución de más pruebas moleculares asegura buen control de la pandemia ^29^. 

Los datos genómicos para agentes patógenos circulantes, en varios países latinoamericanos, eran escasos o inexistentes; con la llegada del SARS-CoV-2, la situación cambió radicalmente, pero la información disponible aún resulta insuficiente ^30^. 

## CONCLUSIONES

En la actual pandemia de la COVID-19, la implementación de pruebas moleculares y participación de laboratorios especializados en identificar nuevos virus, como el SARS-CoV-2 han resultado indispensables para controlar su impacto en salud pública. Al inicio de la pandemia, el LRNVR era el único en el país para la detección molecular de nuevos virus respiratorios como parte de la vigilancia laboratorial de influenza y OVR, con una capacidad inicial de procesamiento de 500 pruebas moleculares diarias, que aumentó progresivamente hasta más de 10 000 pruebas moleculares diarias en la actualidad.

 En el 2019 eran tres los LRR capacitados por TT, para realizar pruebas moleculares de diagnóstico de virus respiratorios dentro de la vigilancia de Influenza y OVR. Actualmente, por necesidad de respuesta ante la pandemia, son 28 los LRR los que han incorporado métodos moleculares a sus capacidades diagnósticas, mediante procesos acelerados de TT. En el decenio pasado, durante la pandemia de la influenza A(H1N1)pdm09, el INS adquirió equipos y mejoró sus capacidades para realizar secuenciamientos genómicos. En la actualidad, la vigilancia del SARS-CoV-2 involucra, de rutina, el análisis genómico por secuenciamiento molecular para evidenciar cambios en las variantes del virus pandémico.

El fortalecimiento e innovación de avances biotecnológicos para la vigilancia basada en laboratorio, con recursos propios y con apoyo externo, y el incremento de capacidades en la RNL, ha permitido generar información oportuna y de calidad para la confirmación diagnóstica durante la actual pandemia. Considerando las diversas realidades regionales y las complejas interacciones interinstitucionales, resulta necesario consolidar este sistema de manera colaborativa y coordinando entre componentes. La infraestructura, recursos y experiencia acumulada en los últimos veintidós años para desarrollar métodos de diagnóstico molecular en el Perú, como parte de la vigilancia laboratorial de influenza y OVR, ha servido de base para el actual monitoreo laboratorial del SARS-CoV-2.
